# Revision of the genus *Tarema* Schaus, 1896 (Lepidoptera, Mimallonoidea, Mimallonidae) with the description of a new species from southeastern Brazil

**DOI:** 10.3897/zookeys.646.10897

**Published:** 2017-01-19

**Authors:** Ryan A. St Laurent, Daniel Herbin, Carlos G. C. Mielke

**Affiliations:** 1 McGuire Center for Lepidoptera and Biodiversity, Florida Museum of Natural History, University of Florida, 3215 Hull Road, Gainesville, FL 32611-2710 USA; 2 7, Le Clos de Lutché, F-31380 Garidech, France; 3 Caixa Postal 1206, 84.145-000 Carambeí, Paraná, Brazil

**Keywords:** Distribution, Neotropical, Paraguay, Tarema bruna sp. n., Tarema fuscosa, Tarema macarina syn. n., Tarema rivara, taxonomy

## Abstract

The genus *Tarema* Schaus, 1896 is revised. The species *Tarema fuscosa* Jones, 1908 and *Tarema rivara* Schaus, 1896 are redescribed, the female of the former is described and figured for the first time, and the genitalia of both sexes for each species are figured for the first time. The lectotype of *Tarema macarina* Schaus, 1928, **syn. n.** is determined to be the female of *Tarema rivara*. *Tarema bruna* **sp. n.** is described from São Paulo, Brazil. Lectotypes for *Tarema fuscosa*, *Tarema rivara*, and *Tarema macarina* are here designated.

## Introduction

The genus *Tarema* Schaus, 1896 has been mostly overlooked in the literature since Schaus (1928), save for its mention in two published species lists of Mimallonidae (Gaede 1931, Becker 1996). Recently, however, Herbin and Mielke (2014) reported new collecting data for *Tarema rivara* Schaus, 1896 quite distant from its type locality, displaying the broad distribution of this species. Additionally, Diniz et al. (2013) reported the first life history information for the genus in reference to *Tarema rivara*, including images of the larva and larval sack, as well as host plant data and the first figures accurately depicting both sexes of this species.

Since Schaus (1928), three species have been included in the genus *Tarema*: *Tarema rivara*, *Tarema fuscosa* Jones, 1908, and *Tarema macarina* Schaus, 1928. Diniz et al. (2013), in figuring both sexes of *Tarema rivara*, unknowingly revealed the conspecifity of *Tarema rivara* and *Tarema macarina*, names applied to the opposite sexes of a single species. We were previously aware of this taxonomic issue, therefore we revise the synonymy and provide accurate figures attributed to the species for aid in future identification. We also provide a distribution map and genitalia figures of both sexes of both species for the first time. Furthermore, a new species is described and figured.

## Methods

Dissections were performed as in Lafontaine (2004). Morphological, including genitalia, terminology follows Kristensen (2003). Not all genitalia were prepared on slides to allow for three-dimensional analysis of the complex male genitalia. Genitalia and abdomens, when not slide mounted, are preserved in glycerol filled microvials.

The primary types (when abdomen was present) and at least one specimen from most localities were dissected.

Specimens from the following collections were examined:

CDH
 Coll. Daniel Herbin, Garidech, France 


CGCM
 Coll. Carlos G. C. Mielke, Curitiba, Paraná, Brazil 


CNC
 Canadian National Collection of Insects, Arachnids and Nematodes, Ottawa, Ontario, Canada 


CPAC
 Coll. Embrapa Cerrados, Planaltina, Distrito Federal, Brazil 


CUIC
Cornell University Insect Collection, Ithaca, New York, USA 


DZUP
 Coll. Pe. Jesus S. Moure, Departamento de Zoologia, Universidade Federal do Paraná, Curitiba, Paraná, Brazil 


MNHU
Museum für Naturkunde der Humboldt-Universität zu Berlin, Germany 


NHMUK
Natural History Museum, London, U.K. 


USNM
National Museum of Natural History [formerly United States National Museum], Washington D.C., USA 


Figures were manipulated with Adobe Photoshop CS4 (Adobe 2008). Male genitalia are figured in natural color with CS4 “auto color” used to improve white backgrounds. Female genitalia were treated with “auto tone” in CS4 to darken characters. The map was created with SimpleMappr (Shorthouse 2010) and edited with CS4. All geographical coordinates are approximate, and are based on the localities provided on specimen labels. GPS data were acquired with Google Earth.

## Results and discussion

### Tarema

Taxon classificationAnimaliaLepidopteraMimallonidae

Schaus, 1896: 55

#### Type species.

*Tarema rivara* Schaus, 1896: 55, by original designation.

#### Diagnosis.

The genus *Tarema* is recognized among the family Mimallonidae by generous amounts of light gray scales present over the entirety of the dorsum and ventrum of the wings, as well as on the thorax and abdomen, giving the species of this genus a hoary appearance. The genitalia of *Tarema* are unique in the family. Male genitalia have short, ovoid valves and spike-covered projections emanating from near the base of the valves that may be associated with the transtilla and/or the gnathos. The gnathos itself is reduced to a flat, movable plate that covers the base of the uncus. Long setae emanate from above the phallus in paired, horsetail-like bunches. The phallus is thick and broad, and has two lengthwise processes terminating in a sharp tip and a curved tip respectively. Female genitalia are robust structures with a medium to large coiled ductus-corpus bursae complex. The sclerotized portion of abdominal segment VIII is broad; appearing wrinkled ventrally, and is covered in thick, branched setae. The genus *Alheita* Schaus, 1928 is somewhat similar to *Tarema* in overall small size, wing shape, and minor resemblance of male genitalia, namely the ovoid valves and odd shape of the uncus which is usually more deeply bifid in *Alheita*.

#### Description.

**Male.** *Head*: Eyes large, more than two thirds area of head; antenna bipectinate to tip, though pectination reduced along distal fifth of antennal length; labial palpus reduced, three segmented, palpus usually not extending beyond frons, scales generally darker brown dorsally. *Thorax*: Appearing hoary due to banded gray or pale khaki scales interspersed amongst darker ones, prothorax with more heavily concentrated light gray or khaki scales. *Legs*: Coloration as for thorax, though lighter gray, vestiture finer, bushier. Tibial spurs narrow, sharp, mostly clothed in scales. *Forewing dorsum*: Forewing length: 9–16 mm, wingspan: 21.5–32.0 mm. Short, triangular, outer margin nearly straight but slightly convex mesally. Ground color ranging from brown or pale clay-orange to nearly black, overall generously shaded by cream or gray scales giving the wing a hoary, layered appearance. Antemedial line absent or nearly so, faint dark band may be present. Postmedial line nearly straight but may be somewhat inwardly or outwardly bent, line preapical such that submarginal area mostly uniform in width from tornus to apex. Apical half of submarginal area with postmedial lunule. Costa appearing lighter than most of wing due to high concentration of gray or khaki scales. Discal spot a thick streak spanning width of discal cell. *Forewing ventrum*: Antemedial line absent, postmedial line never straight, bulging outward toward wing margin mesally. *Hindwing dorsum*: Submarginal area with orange to reddish patch of scales mesally, discal mark present but smaller. *Hindwing ventrum*: Following same pattern as forewing ventrum. Frenulum as single bristle. *Venation*: Rather typical for Mimallonidae, discal cell quite broad, distal edge sharply slanted (see Schaus 1896). *Abdomen*: Short, barely or not extending beyond anal angle of hindwing. *Genitalia*: Complex; vinculum somewhat ovoid or almost circular, ventrally with reduced saccus. Uncus robust, sharp, parrot beak-like and dorsolaterally flattened or reduced to slightly triangular stump with slight bidentation terminally. Gnathos a flattened or curved plate concealing fingerlike sclerotization of anal tube. Valves short, rounded, weakly sclerotized mesally. Base of valves with pair of fingerlike projections, weakly sclerotized knobby area may be present above fingerlike projections. Valves with or without more heavily sclerotized, spined accessory arms that may or may not be attached basally to valves. Diaphragm with pair of horsetail-like seatal patches consisting of setae of variable length that extend outward over phallus below gnathos plate. Juxta partially fused to phallus, encircling it, lightly sclerotized, with ventral lip connecting phallus to base of vinculum (severed to excise phallus). Phallus broad, large, with two elongated accessory projections. Vesica balloon-like, slightly scobinate, separated into fairly distinct diverticula. **Female.** *Head*: Similar to male, but broader, antennae and labial palpi smaller. *Thorax*: As in male. *Legs*: As in male, though tibial spurs thicker. *Forewing dorsum*: Forewing length: 10.0–18.5 mm, wingspan: 22.0–34.5 mm. As in male but slightly broader, postmedial line usually more noticeably bent. *Forewing ventrum*: Similar to forewing ventrum of male, but veins usually lined with contrasting yellow scales. *Hindwing dorsum*: Coloration and markings as for forewing dorsum. *Hindwing ventrum*: Follows same pattern as forewing ventrum. Frenulum absent. *Abdomen*: As in male but slightly more robust. Tergite VIII as three posteriorly directed lobes or as single broad plate, sternite VIII as wrinkled mass consisting of one or two pieces, covered in thick, branched setae. *Genitalia*: Stout, robust or quite narrow overall; apophyses anteriores highly reduced, apophyses posteriores elongate, spanning length of segment IX. Lamella indistinct due to large sclerotization of sternite VIII. Corpus bursae large, bag-like, coiled, broadly connected beneath sternite VIII /ostium complex, no clear ductus bursae present, occasionally with large, snake-like spermatophore present within corpus bursae. Papillae anales typical of Mimallonidae, appearing rectangular laterally; papillae anales covered with fine setae.

#### Key to species of Tarema*

**Table:** 

1	Male: coloration mostly clay-brown or orange to more red with abundant gray shading. Valves with heavily sclerotized, flattened, spined structures, or no accessory arms. Phallus with smooth dorsal projection. Female: mostly brown and gray, but may have clay or salmon-orange hue, especially antemedially, darker apical patch absent, tergite VIII trilobed	**2**
-	Male: coloration mostly dark brown, to nearly black with cream colored shading; valves with heavily sclerotized, tubular, clubbed spine structures; phallus with spined dorsal projection. Female: mostly dark brown to black with gray shading, tergite VIII as singular plate	***Tarema fuscosa***
2	Ground color clay-orange to orange-red, postmedial lunule angled away from postmedial line toward wing margin, becoming diffuse before reaching wing margin. Male genitalia with heavily sclerotized, flattened, spined structures, connected lengthwise to short, rounded valve, gnathos as rectangular plate, dorsal phallus projection broad. Female: tergite VIII with trilobed plate	***Tarema rivara***
–	Ground color earthen brown, postmedial lunule parallels postmedial line until bending toward wing margin, reaching it. Male genitalia lacking large, heavily sclerotized vincular/valve arms, which are instead reduced to small sclerotizations at base of valve, gnathos as tapered hexagonal plate, dorsal phallus projection thin	*Tarema bruna* sp. n.*

*The female of *Tarema bruna* sp. n. is unknown.

### Tarema rivara

Taxon classificationAnimaliaLepidopteraMimallonidae

Schaus, 1896

Figs 1–6 , 11 , 14 , 16

Tarema rivara Schaus, 1896: 55
Tarema rivara ; Schaus 1928: 670, fig. 88i ♂
Tarema rivara ; Gaede 1931
Tarema rivara ; Becker 1996
Tarema rivara ; Diniz et al. 2013: 88, figs ♂, ♀, larva, larval sack
Tarema macarina Schaus, 1928: 670, fig. 88i ♀ **syn. n.**
Tarema macarina ; Gaede 1931
Tarema macarina ; Becker 1996

#### Type material.

***Tarema rivara* Schaus: lectotype [here designated**], ♂. **BRAZIL: São Paulo**: São Paulo, S.E. Brazil./ Collection, WmSchaus/ Type No. 12566 U.S.N.M/ USNM-Mimal: 1120/ *Tarema rivara* Type. Schaus./ LECTOTYPE male *Tarema rivara* designated by St Laurent, Herbin, and C. Mielke, 2017 [handwritten red label]/ St Laurent diss.: 8-22-16:3/ (USNM, examined). Type locality: Brazil: São Paulo.

***Tarema macarina* Schaus: lectotype [here designated**], ♀. **BRAZIL: São Paulo**: 1 ♀, São Paulo, S.E. Brazil./ Collection, WmSchaus/ Type No. 33596 U.S.N.M/ USNM-Mimal: 1121/ *Tarema macarina* Type Schaus/ LECTOTYPE female *Tarema macarina* designated by St Laurent, Herbin, and C. Mielke, 2017/ *Tarema rivara* det. St Laurent 2016/ St Laurent diss.: 8-22-16:2/ (USNM, examined). **Paralectotypes**, 2 ♀. **BRAZIL: São Paulo**: 1 ♀, Brasil. m., S. Paulo 96/ Typus/ Coll. Staudinger/ *Tarema macarina* Schaus, co-type/ (MNHU, photograph examined). **Unknown state**: 1 ♀, Brasil [illegible]/ *Tarema macarina* co-type Schaus/ 8582/ (MNHU, photograph examined). Type locality: Brazil: São Paulo. – All paralectotypes with the following yellow label: PARALECTOTYPE ♀ *Tarema macarina* designated by St Laurent, Herbin, and C. Mielke, 2017.

#### Additional specimens examined.

(28 ♂, 14 ♀) **BRAZIL: Maranhão**: 1 ♂, Feira Nova do Maranhão, Retiro, 07°00'31"S, 46°26'41"W, 480 m: 16–17.II.2013, C. Mielke leg., Coll. C. Mielke 26.333 (CGCM). **Bahia**: 1 ♂, Barreiras, 12°9'S, 45°00'W, 700 m: 4.II.1994, Coleção EMBRAPA-CPAC No. 15.987, [Camargo leg.] (CPAC). **Mato Grosso**: 1 ♀, Chapada [dos] Guimarães: 25.V.1989, V.O. Becker leg., Coll. Becker 75031, USNM-Mimal: 2320 (USNM). 1 ♂, No specific locality: XII.1929, Coll. R. Spitz, Rothschild Bequest 1939–1 (NHMUK). **Goiás**: 1 ♂, Ponte Funda, Vianópolis: 24.X.1987, Tangerini leg., genitalia prep. D. Herbin ref H. 1010 (CDH). 1 ♀, Ipameri: 10.X.1988, V.O. Becker leg., Coll. Becker 59419, USNM-Mimal: 2323, St Laurent diss.: 8-22-16:8 (USNM). **Distrito Federal**: 1 ♂, 1 ♀, Estação Florestal, Cabeça do Veado, 1100 m: 17.X.1971, 23.X.1971, E.G., I. & E.A. Munroe leg., St Laurent diss.: 3-14-16:7, 3-14-16:8 (CNC). 5 ♂, 2 ♀, Planaltina, 15°35'S, 47°42'W, 1000 m: 1–10.XI.1994, Tangerini leg. (1 ♂, CDH); 25.IX.1985, 5.XI.1988, V.O. Becker leg., Coll. Becker 57771, 58871, 58872, USNM-Mimal: 2301–2304, 2321, 2322 (4 ♂, 2 ♀, USNM). 1 ♂, Planaltina: 15.X.1995 (MWM). **Minas Gerais**: 1 ♂, Serra do Cipó à Conceição do Mato Dentro, km 126.3, 19°14'51"S, 43°30'38"W, 1270 m: 18.XI.2012, genitalia prep. D. Herbin ref H. 1012 (CDH). 1 ♂, Malacacheta, 500 m: I.1998, H. Thöny leg., genital prep. 29.234 (MWM). 1 ♂, Sete Lagoas, 720 m: 15.III.1974, V.O. Becker leg., Coll. Becker 411, USNM-Mimal: 2300 (USNM). 1 ♂, Paracatu, 17°13'S, 46°52'W, 920 m: 5.II.1994, Coleção EMBRAPA-CPAC No. 14.664 (CPAC). 1 ♂, Iraí de Minas, 18°43'S, 47°30'W, 950 m: 9.II.1994, Coleção EMBRAPA-CPAC No. 14.145 (CPAC). 1 ♂, São Roque de Minas, São José do Barreiro, 870 m, 3.XII.2016, C. Mielke leg., Coll. C. Mielke 32.162 [point not on map] (CGCM). **São Paulo**: 2 ♂, Ribeirão Preto, Fazenda da Pedra, Rio Tamanduá: Travassos & Pearson leg., 12–15.X.1953 (NHMUK); HRP 643, USNM-Mimal.: 2425 (USNM). 1 ♂, Locality as for previous but 500 m: 2–5.III.1954, Pearson & Oiticica leg., Brit. Mus. 1962–112 (NHMUK). 1 ♂, Alto da Serra [Paranapiacaba]: I.1926, R. Spitz leg., Rothschild Bequest 1939–1, St Laurent diss.: 7-7-16:4 (NHMUK). 1 ♂, Miracatu, 700 m: 20.XI.1997, H. Thöny leg., genital prep. 29.233 (MWM). 2 ♂, 2 ♀, No additional locality data: E.D. Jones Coll., Brit. Mus. 1919–295 (NHMUK); Rothschild Bequest 1939–1 (NHMUK). 1 ♂, No specific locality, 750 m: E.D. Jones Coll., Brit. Mus. 1919–295, NHMUK010354581, St Laurent diss.: 8-29-16:5 (NHMUK). **Paraná**: 1 ♂, Campo do Tenente, 850 m: 17.X.1985, [O.] Mielke leg., DZ 15.496 (DZUP). 1 ♂, Ponta Grossa: II.1957, at light, No. 1780, Coll. F. Justus Jor (DZUP). 1 ♀, Castro, 950 m: E.D. Jones leg., E.D. Jones Coll., Brit. Mus. 1919–295 (NHMUK). 1 ♀, Tucunduva [Sengés], 650 m: 17.II.1913, E.D. Jones leg., E.D. Jones Coll., Brit. Mus. 1919–295 (NHMUK). **Santa Catarina**: 1 ♂, No additional locality data: F. Hoffmann leg., USNM-Mimal: 2681, St Laurent diss.: 8-22-16:7 (USNM). **No state**: 1 ♀, “Bresil”, Joicey Coll. Brit. Mus. 1925–157 (NHMUK). **PARAGUAY: Cordillera**: 1 ♀, Pirareta, 25°29'S, 56°56'W, 200 m: 26–31.VIII.2012, [U. Drechsel] leg., genitalia prep. D. Herbin ref H. 1014 (CDH). **Amambay**: 1 ♀, Estancia Oliva, 22°10'S, 56°26'W, 225 m: 23–25.I.2013, [U. Drechsel leg.], (CDH). **Guairá**: 1 ♀, Villarica: 2.X.1925, F. Schade leg., J.J. Joicey Esq., B.M. 1929–458, St Laurent diss.: 7-7-16:5 (NHMUK). **Paraguarí**: 1 ♀, Sapucay [*recte* Sapucai]: 12.VIII.1904, W. Foster leg., Rothschild Bequest 1939–1 (NHMUK).

#### Diagnosis.

*Tarema rivara* can be differentiated from others in the genus by the pervasive orange to orange-red coloration on the wings in males, and by the faint orange hue on the wings of the females which is concentrated antemedially and/or submarginally. The more similar *Tarema bruna* sp. n. is more earthen brown in color than *Tarema rivara*, with a longer postmedial lunule that reaches the wing margin without becoming diffuse. In both sexes, *Tarema rivara* has a smaller wingspan than *Tarema fuscosa* and lacks a distinct dark brown patch of scales at the apex of the forewings. This patch of scales is distinct in *Tarema fuscosa* due to the contrast with the light cream color of the postmedial lunule that borders it. Genitalia are quite different between these species, in *Tarema rivara* the valves are reduced to small lobes connected to a flattened, spiny accessory, while the valves of *Tarema fuscosa* and *Tarema bruna* sp. n. are larger and not connected to, or lack the accessory arms. The gnathos of *Tarema rivara* is a rectangular plate rather than ovoid as in *Tarema fuscosa*, or tapered hexagonal as in *Tarema bruna* sp. n., the fingerlike projections at the base of the valves are smaller in *Tarema rivara*, and finally the phallus of *Tarema rivara* (and *Tarema bruna* sp. n.) bears a smooth dorsal projection whereas the same projection is short and spined in *Tarema fuscosa*. The key differences in female genitalia are the larger corpus bursae in *Tarema rivara* and the trilobed tergite VIII, which is a broad, singular plate in *Tarema fuscosa*. The female tergite can usually be examined under a microscrope after brushing off scales, without dissecting the specimen.

#### Description.

**Male.** *Head*: As for genus, gray with orange undertone, antenna coloration as for head. *Thorax*: Coloration similar to that of head, but more orange, appearing hoary due to banded gray scales interspersed amongst orange hued ones, prothorax covered almost entirely in light gray scales. *Legs*: As for genus but tibia mostly orange. *Forewing dorsum*: Forewing length: 9–13 mm, avg.: 11.5 mm, wingspan: 21.5–29.0 mm, n=16. Ground color ranging from clay-orange to almost brick red, overall generously shaded by gray scales giving the wing a hoary, layered appearance, especially medially. Postmedial line as for genus but coloration light cream and bordered externally with black scaling continuously along length. Antemedial area with salmon orange hue, medial area always lighter gray compared to orange or reddish submarginal area. Apical half of submarginal area with postmedial lunule, the latter never parallel with margin or postmedial line, either smoothly curved toward margin or angled acutely from postmedial line, becoming diffuse before reaching wing margin, basal half of submarginal area with bright orange or red patch along postmedial line. Discal spot as for genus. Fringe light gray to khaki with lighter and darker patches, including salmon colored scales. *Forewing ventrum*: Similar to dorsum but usually lighter due to more extensive covering of gray scales; antemedial line absent, postmedial line very faint, bulging outward toward wing margin mesally. Postmedial lunule present as on dorsum, more distinct than postmedial line. *Hindwing dorsum*: Coloration as for forewing dorsum, following similar patterning but antemedial line absent, postmedial line slightly concave, and submarginal area always orange to more reddish, postmedial lunule absent or just a faint suffusion, coloration usually concentrated somewhat mesally. *Hindwing ventrum*: Following same pattern as forewing ventrum. *Abdomen*: As for genus, concolorous with thorax. *Genitalia*: (Fig. 11) n=12. Vinculum almost circular. Uncus robust, sharp, parrot beak-like when viewed laterally, uncus dorsolaterally flattened. Gnathos a rectangular, elongated plate with slight curvature mesally. Valve short, rounded, weakly sclerotized mesally, strongly affixed to vinculum such that they do not open fully. Base of valves with pair of small, fingerlike projections, weakly sclerotized knobby area present above fingerlike projections. Valve with more heavily sclerotized, spined accessory arms attached basally to valves, arms originate from transtilla or base of valve (unclear), connected along length of valve. Accessory arms flattened and ventrally spined. Diaphragm with pair of horsetail-like seatal patches consisting of setae of variable length that extend outward over phallus for about three-quarters length of gnathos plate, setae mostly straight. Phallus broad, large, with two elongated accessory projections of variable length, projection superior to phallus smooth, straight, sharply pointed; other projection shorter, narrower, running laterally along phallus originating from within phallus, tip of second projection sharp, but variously bent, size of phallus relative to projections somewhat variable. Vesica balloon-like, slightly scobinate. **Female.** *Head*: Similar to male, but broader, antennae and labial palpi smaller. *Thorax*: As in male, but usually grayer or occasionally more salmon colored. *Legs*: As in male, though tibial spurs thicker. *Forewing dorsum*: Forewing length: 10–15 mm, avg.: 12.1 mm, wingspan: 22–30 mm, n=13. Sexual dimorphism strong, wing shape and markings similar to male, but wing broader, coloration usually much more subdued gray and brown, if salmon hue present, generally restricted to antemedial area, though rarely some specimens with salmon hue more pervasive, especially submarginally, otherwise submarginal area solid brown, postmedial lunule usually fainter than in male, sometimes almost absent except for small streak. Fringe with distinctly black portion apically. *Forewing ventrum*: Similar to forewing ventrum of male, but salmon hue generally absent, veins usually lined with contrasting yellow scales, wing grayer overall but apical region more distinctly solid brown than in male. *Hindwing dorsum*: Coloration and markings as for forewing dorsum, though postmedial lunule absent. *Hindwing ventrum*: Follows same pattern as forewing ventrum. *Abdomen*: As in male but slightly more robust, coloration subdued. Tergite VIII as three posteriorly directed lobes, sternite VIII as large, curved, wrinkled mass, covered in thick, branched setae. *Genitalia*: (Fig. 14) n=5. As for genus, two dissections with large, snake-like structure, apparently a spermatophore based on presence in one dissected male, present within corpus bursae. Papillae anales narrow.

**Figures 1–6.**
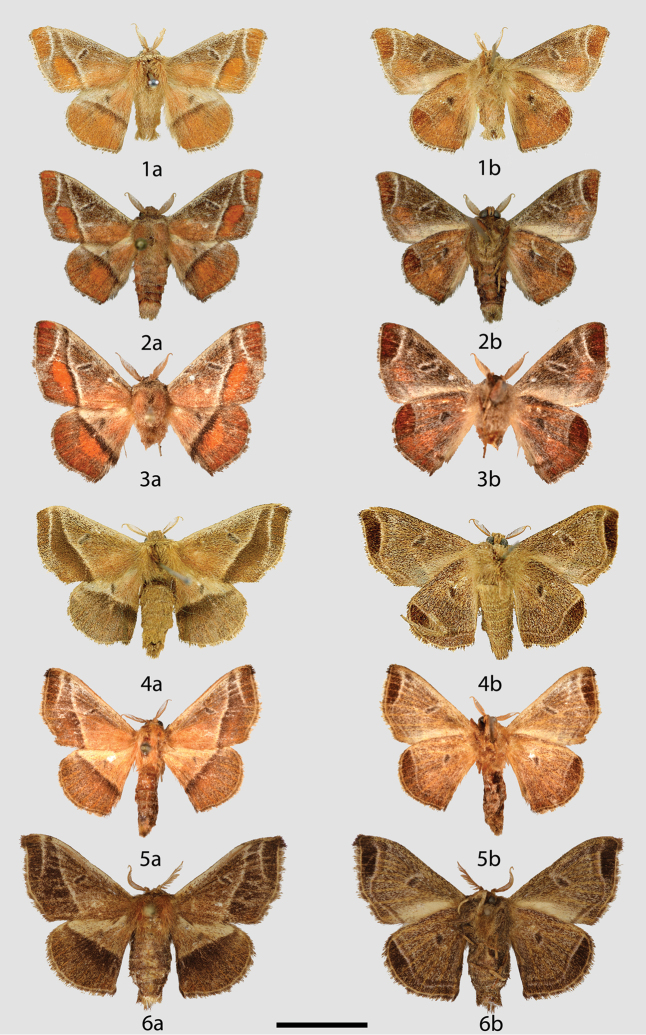
*Tarema rivara* adults, **a** dorsal **b** ventral. **1** ♂, Lectotype, Brazil, São Paulo (USNM) **2** ♂, Brazil, Maranhão, Feira Nova do Maranhão, Retiro, 480 m [image originally published by Antenor, reused with permision] (CGCM) **3** ♂, Brazil, Minas Gerais, Serra do Cipó à Conceição do Mato Dentro, km 126.3, 1270 m (CDH) **4** ♀, Lectotype of *Tarema macarina* syn. n., Brazil, São Paulo (USNM) **5** ♀, Paraguay, Amambay, Estancia Oliva, 225 m (CDH) **6** ♀, Brazil, Distrito Federal, Estação Florestal, Cabeça do Veado, 1100 m (CNC). Scale bar: 1 cm.

**Figures 7–10.**
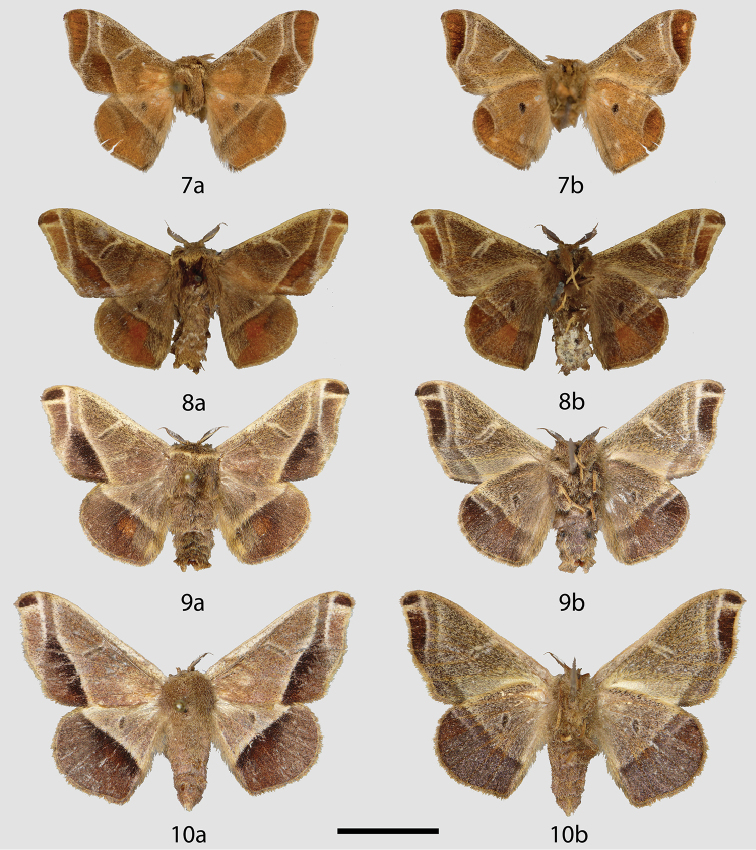
*Tarema* adults, **a** dorsal **b** ventral. **7** *Tarema bruna* holotype ♂, Brazil, São Paulo, Alto da Serra [Paranapiacaba] (NHMUK) **8** *Tarema fuscosa* lectotype ♂, Brazil, Paraná, Castro (NHMUK) **9** *Tarema fuscosa* ♂, Brazil, São Paulo, Guapiara, Paivinha, 800 m (CGCM) **10** *Tarema fuscosa* ♀, Brazil, São Paulo, Guapiara, Paivinha, 800 m (CGCM). Scale bar: 1 cm.

**Figures 11–13.**
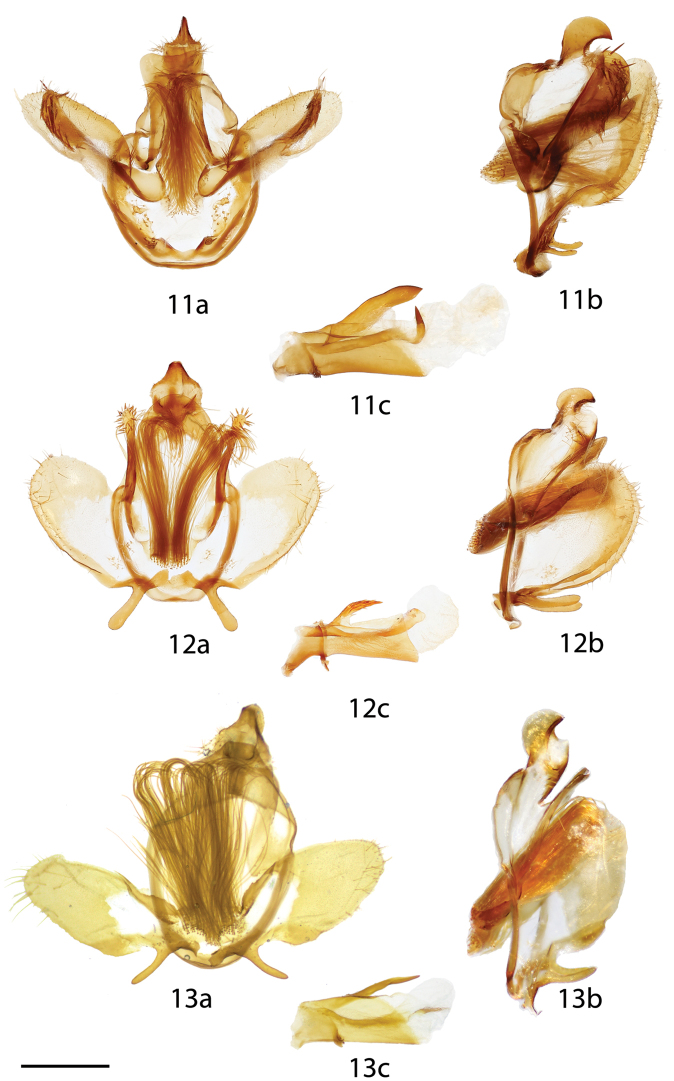
*Tarema* male genitalia, **a** ventral **b** lateral **c** phallus lateral. **11** *Tarema rivara*, Brazil, Distrito Federal, Estação Florestal, Cabeça do Veado, 1100 m, St Laurent diss.: 3-14-16:7 (CNC) **12** *Tarema fuscosa*, Brazil, Santa Catarina, St Laurent diss.: 2-26-16:7 (CUIC) **13** *Tarema bruna* holotype, Brazil, São Paulo, Alto da Serra [Paranapiacaba], NHMUK010402168 genitalia prep. [13b horizontally flipped] (NHMUK). Scale bar: 1 mm.

**Figures 14, 15.**
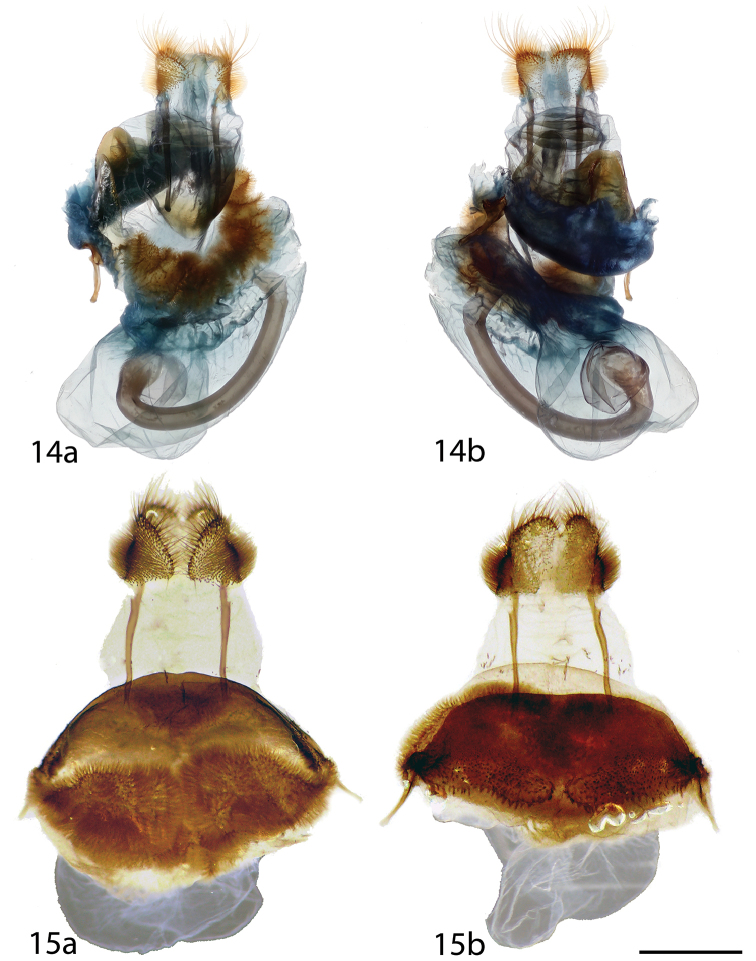
*Tarema* female genitalia, **a** ventral **b** dorsal. **14** *Tarema rivara*, Brazil, Distrito Federal, Estação Florestal, Cabeça do Veado, 1100 m, St Laurent diss.: 3-14-16:8 (CNC) **15** *Tarema fuscosa*, Brazil, São Paulo, Guapiara, Paivinha, 800 m, C. Mielke genitalia prep. CGCM 26.955 (CGCM). Scale bar: 1 mm.

#### Distribution

**Figure 16.**
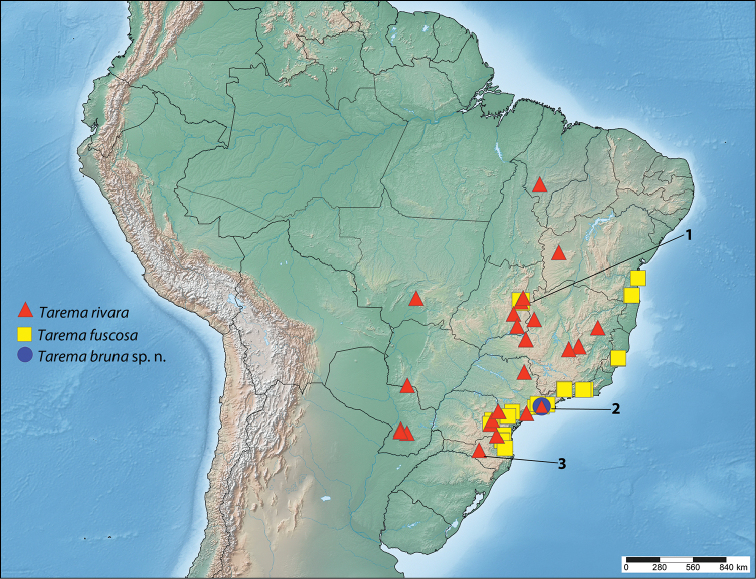
Known distribution of *Tarema*. Numbers superimposed on the map refer to the following annotations: **1** Outlier data point for *Tarema fuscosa* in Brazil, Distrito Federal may be erroneous (see remarks for that species). **2** The point where all three symbols are found on top of each other is a single locality, that being Brazil, São Paulo, Paranapiacaba. **3** Data point for *Tarema rivara* in Brazil, Santa Catarina is placed at the center of the state because no detailed locality data is available for this species from Santa Catarina.

**(Fig. 16).** This species has a wide distribution in South America, and although most records come from Brazilian Cerrado in Bahia, Maranhão, Mato Grosso, Goiás, Minas Gerais, and Distrito Federal, *Tarema rivara* is also known from Brazilian Atlantic Forest in São Paulo, mixed ombrophilous forest in the state of Paraná, and inland forests of Paraguay. We are also aware of one specimen from Santa Catarina, though unfortunately it lacks more detailed data that would allow us to understand the habitat in which it inhabits in this state.

#### Natural history.

Diniz et al. (2013) report *Tarema rivara* larvae feeding on Vochysiaceae, including the species *Qualea grandiflora*, *Qualea multiflora*, and *Qualea parviflora*. The same authors describe the larval sack as being constructed from leaves, silk, and feces. These larval sacks are of the less uniformly constructed variety in Mimallonidae, and are thus more similar to those of *Lacosoma* Grote, 1864 and *Cicinnus melsheimeri* (Harris, 1841) rather than highly compacted, rigid structures as seen in *Menevia* Schaus, 1928, *Cicinnus packardii* (Grote, 1865), or *Cicinnus bahamensis* St Laurent & McCabe, 2016, among others (R. A. St. Laurent pers. obs.).

#### Remarks.

In the original description of *Tarema rivara*, Schaus (1896) stated: “What I believe to be the ♀ of this species has the reddish shades replaced by dark brown.” Apparently Schaus was well aware of the dimorphism of *Tarema rivara* at the time of its original description, thus it is somewhat surprising that he described the female of *Tarema rivara* as a new species, *Tarema macarina*, over 30 years later (Schaus 1928). We are also aware of a *Tarema rivara* female specimen determined as this species and not *Tarema macarina* at the NHMUK. However, we infer that Schaus changed this earlier determination due to the fact that he had located what he believed to be the male of *Tarema macarina* in the MNHU as per the following statement from Schaus (1928) in his treatment of *Tarema macarina*: “A male of this species is in the Berlin museum.” Two syntypes of *Tarema macarina* are present in the MNHU with Schaus’s handwriting on the “type” labels; however, both specimens are female, as is the lectotype (here designated) in the USNM.

The complete lack of any male specimens correctly determined as *Tarema macarina* and the unusual disparity of female *Tarema rivara* led us to believe that these names are synonyms. Many records of *Tarema rivara* and *Tarema macarina* are sympatric, and thus support this hypothesis. A close analysis of *Tarema rivara* females reveals hints of orange coloration antemedially, a coloration abundant in the male specimens, but not in *Tarema fuscosa*. Given that *Tarema fuscosa* females can be easily determined as such due to the lack of sexual dimorphism in this species, the disparity of opposite sexes for *Tarema rivara* and *Tarema macarina* provides clear evidence that they represent a single, dimorphic species. Furthermore, a dissection of *Tarema rivara* females reveal the long, snake-like spermatophore seen in male *Tarema rivara*, unlike the smaller spermatophore of *Tarema fuscosa*.

Diniz et al. (2013) figure a larva and adults of both sexes of *Tarema rivara*, correctly figuring the female of *Tarema rivara*, which again, matches the type specimens of *Tarema macarina*.

Compared to *Tarema fuscosa* below, this species seems to primarily be an inhabitant of drier Cerrado but is also present in the more humid Atlantic forest in the states of São Paulo (type locality) and Paraná where it is sympatric with *Tarema fuscosa*, but apparently not synchronic. In these regions of sympatry, *Tarema rivara* flies during the summer (October through February), while *Tarema fuscosa* flies in the winter, though exceptions to these flight times are present in regions where both species are not found together.

### Tarema fuscosa

Taxon classificationAnimaliaLepidopteraMimallonidae

Jones, 1908

Figs 8–10 , 12 , 15 , 16

Tarema fuscosa Jones, 1908: 173–174
Tarema fuscosa ; Schaus 1928: 670, fig. 88i ♂
Tarema fuscosa ; Gaede 1931
Tarema fuscosa ; Becker 1996

#### Type material.

**Lectotype [here designated**], ♂. **BRAZIL: Paraná**: Castro, Paraná, 950 m, E.D. Jones / *Tarema fuscosa* Type D. Jones/ E.D. Jones Coll., Brit. Mus., 1919–295/ BMNH(E) #805428/ SYN-TYPE/ NHMUK010354542/ [genitalia] VIAL NHMUK010402134/ LECTOTYPE male *Tarema fuscosa* designated by St Laurent, Herbin, and C. Mielke, 2017 [handwritten red label]/ (NHMUK, examined). Type locality: Brazil: Paraná: Castro.

#### Additional specimens examined.

(114 ♂, 4 ♀) **BRAZIL: Distrito Federal**: 1 ♂, Estação Florestal, Cabeça do Veado, 1100 m: 17.X.1971, E.G., I. & E.A. Munroe leg., St Laurent diss.: 3-14-16:10 (CNC). 2 ♂, Parque do Gama: 10.X.1971, E.G. Munroe & K.S. Brown leg., St Laurent diss.: 3-14-16:9 (CNC). **Bahia**: 35 ♂, 1 ♀, env. Camacan (SB), 15°25'S, 39°34'W, 800m: X.2011, XI.2011, XII.2011, H. Thöny leg., genital prep. 29.217 (MWM). 1 ♂, env. Camacan (SB), 750 m: IV.2011, H. Thöny leg. (MWM). 1 ♂, 1 ♀, env. Camacan, ca. 750 m: 10–14.XI.2010, Th. Greifenstein leg. (MWM). 2 ♂, Camacan, 15°24'S, 39°30'W: III.2011, H. Thöny leg. (MWM). 1 ♂, Maraú, Fazenda Água Boa, 14°13'S, 39°29'W, 150 m: IV.2011, H. Thöny leg. (MWM). 2 ♂, env. Camacan, 15°25'S, 39°34'W, 800 m: X.2012, H. Thöny leg. (MWM). **Espírito Santo**: 10 ♂, Santa Leopoldina, Village Tirol, 24°75'S, 40°50'W, 700 m: 22–31.X.1996, 20.II–30.III.1997, V.1997, 15.V.1997, VIII.1997, 15.IX.1997, VI.1998, X.1999, H. Thöny leg. (MWM). 5 ♂, Santa Leopoldina, Village Tirol, 700 m: III.1999, VI.1999, X.1999, III.2000, H. Thöny leg. (MWM). 1 ♂, Santa Leopoldina, Village Tirol, 20°10'S, 40°33'W, 700 m: XI.2000, H. Thöny leg. (MWM). 9 ♂, Santa Leopoldina, Boqueirão, 600 m: 15.II.199, VI.1997, 15.IX.1997, H. Thöny leg. (MWM). 2 ♂, No additional locality data: USNM-Mimal: 2729, 2730 (USNM). **Rio de Janeiro** 1 ♂, Barreira, Teresópolis: 18.X.1955, Coll. Gagarin (DZUP). 1 ♂, Petrópolis: 19.XI.1928, Gagarin leg., Coll. Gagarin (DZUP). 4 ♂, Parque Nacional do Itatiaia, Lago Azul, 800 m: 20–22.VI.1955, R. Barros, D. Albuquerque, & Pearson leg. (NHMUK). 1 ♂, Itatiaia, Horto Florestal (=horticultural garden), 800 m: 10.VIII.1953, Travassos & Pearson leg., Brit. Mus. 1962-112 (NHMUK). 1 ♂, No additional locality data: Coll. Thalenhorst, Coll. Staudinger (MNHU). **São Paulo**: 9 ♂, 1 ♀, Guapiara, Paivinha, 800 m: 5–6.XI.2004 (1 ♂), 24–27.VII.2005 (2 ♂), 18.VII.2007 (1 ♂), 11.VIII.2007 (3 ♂, 1 ♀), 12.IX.2007 (2 ♂), C. Mielke leg., Coll. C. Mielke 25.772, 26.433, 26.535, 26.537, 26.680, 26.955, 26.977, 27.060, 28.074, 28.106 (CGCM). 1 ♂, Embu-Guaçu: Sitío, L. Travassos F. leg. (CDH). 4 ♂, Apiaí, 750 m: 8.VIII.2006 (2 ♂), 7.IX.2007 (2 ♂), C. Mielke leg. (CDH). 1 ♂, Ypiranga [*recte* Ipiranga, São Paulo]: V.1924, R. Spitz, Rothschild Bequest, BM 1939–1 (NHMUK). 2 ♂, Alto da Serra [Paranapiacaba]: VI.1926, VII.1928, R. Spitz leg., Rothschild Bequest, BM 1939–1 (NHMUK). 2 ♂, Salesópolis, Boracea [Boracéia], 850 m: 26.VIII.1949, Travassos, Travassos Filho, Pearson, & Rabello leg., Brit. Mus. 1962-112 (NHMUK); 23–26.V.1952, Pearson leg., 528, USNM-Mimal: 2424 (USNM). **Paraná**: 2 ♂, Ponta Grossa: IV.1948, No. 1552, Coll. F. Justus Jor (DZUP). 1 ♂, Tijucas do Sul [*recte* Guaratuba], Castelhanos, 20°26'S, 54°39'W [coordinates likely incorrect], 500 m: 1.VI.1999, H. Thöny leg. (MWM). **Santa Catarina**: 1 ♂, São Bento do Sul, Rio Vermelho, Road to Rio Natal, 26°20'00.77"S, 49°18'28.25"W, 503 m: no date, Rank leg., genitalia prep. D. Herbin ref H. 1013 (CDH). 8 ♂, São Bento do Sul, Rio Natal, 850 m: VI.1998, VII.1998, VIII.1998, IX.1998, X.1998, VII.1999, H. Thöny leg. (MWM). 1 ♂, Blumenau: E. Wenzel S.G. leg. (MNHU). 1 ♂, No additional locality data: St Laurent diss.: 2-26-16:7 (CUIC). 1 ♂, No additional locality data/illegible: Dognin Coll., 269, USNM-Mimal: 2678 (USNM). **No state**: 1 ♀, “Brasil”, Mssn. G. (MNHU).

#### Diagnosis.

Compared to the other two species in the genus, *Tarema fuscosa* is easily recognized by the very dark brown to nearly black ground color, with a dark patch at the apex of the forewings surrounded by pale cream markings. The male genitalia is unique in having heavily sclerotized, spiny, club-like vincular arms that are not connected lengthwise to the valves. This is also the only species in the genus with an ovoid gnathos plate. Additionally, the dorsal projection of the phallus is short and spiny, not smooth as in the other two species. The female genitalia have a smaller corpus bursae than in *Tarema rivara* and a broad singular tergite VIII, as opposed to the trilobed corresponding tergite of *Tarema rivara*.

#### Description.

**Male.** *Head*: As for genus, grayish brown; antenna coloration usually as for head, though pectination darker brown than flagellum; labial palpus reduced, apparently three segmented, but third segment much reduced. *Thorax*: Coloration similar to that of head, though appearing hoary due to banded brown and pale khaki to cream colored scales, prothorax with more heavily concentrated khaki or cream colored scales. *Legs*: Coloration as for thorax, though femur and tibia darker brown, tarsus lighter, cream colored. *Forewing dorsum*: Forewing length: 11–16 mm, avg.: 13.8 mm, wingspan: 22–31 mm, n=17. Ground color ranging from pale reddish brown to nearly black, overall generously shaded by cream colored scales giving the wing a hoary, layered appearance. Postmedial line as for genus, but coloration light cream not bordered externally with black except for darkened region concentrated near to tornus. Ante- and median areas usually concolorous, submarginal area with reduced cream colored scales, appearing much darker red-brown, brown, to nearly black. Apical half of submarginal area with postmedial lunule, the latter either slightly curved toward wing margin, or nearly parallel with margin, especially along apical half of lunule, basal half of submarginal area with darker red-brown or black patch along postmedial line, apex with darker brown patch outlined by white lunule and cream colored patch immediately beneath darker apical patch. Costa appearing lighter than most of wing due to heavy concentration of cream or khaki colored scales. Discal spot as for genus. Fringe light gray to khaki with lighter and darker patches. *Forewing ventrum*: Similar to dorsum but usually lighter due to more extensive covering of cream and khaki scales, some of which appear yellowish, apical half of submarginal area darker than that of dorsum, except where interrupted by lighter band below apical patch; antemedial line always absent, postmedial line never straight, angled outward toward wing margin mesally. *Hindwing dorsum*: Coloration as for forewing dorsum, following similar patterning but antemedial line absent, postmedial line slightly concave, and submarginal area more uniformly dark reddish brown, dark brown, or black, always with contrasting orange patch of scales mesally. *Hindwing ventrum*: Following same pattern as forewing ventrum, postmedial lunule reduced to straight, faint streak. *Abdomen*: As for genus, concolorous with thorax. *Genitalia*: (Fig. 12) n=7. Vinculum somewhat ovoid, ventrally with reduced saccus. Uncus robust but reduced to slightly triangular stump with slight bidentation terminally. Gnathos an ovoid, elongated, mesally indented plate. Valves short, rounded, weakly sclerotized mesally. Base of valves with pair of long, fingerlike projections. Valves with more heavily sclerotized, spined accessory arms connected to vinculum. Accessory arms narrow and tube-like, terminating in enlarged club end with spines concentrated terminally or present along entire length of arm. Diaphragm with pair of horsetail-like setal patches consisting of very long setae that extend outward over phallus below gnathos plate, setae curled backward at end. Phallus broad, large, widened distally, with two elongated accessory projections, one projection more variable in length, superior to phallus, irregular, pointed, spined; other projection longer, narrower, running laterally along phallus originating from within phallus, tip of second projection sharp, angled backward. Vesica balloon-like, slightly scobinate, separated into fairly distinct diverticula. **Female.** *Head*: Similar to male, but broader, antennae and labial palpi smaller. *Thorax*: As in male, though cream colored scales may be a bit yellower. *Legs*: As in male. *Forewing dorsum*: Forewing length: 17.0–18.5 mm, avg.: 17.5 mm, wingspan: 31.0–34.5 mm, n=3. Sexual dimorphism reduced, as in male but slightly broader, postmedial line usually more noticeably bent. *Forewing ventrum*: Similar to forewing ventrum of male, but veins usually lined with yellow scales. *Hindwing dorsum*: Coloration and markings as for forewing dorsum, orange mesal patch present in male very faint in female. *Hindwing ventrum*: Follows same pattern as forewing ventrum. *Abdomen*: As in male but slightly more robust. Tergite VIII as single broad plate, sternite VIII as wrinkled mass consisting of two pieces, covered in thick, branched setae. *Genitalia*: (Fig. 15) n=1. As for genus but particularly stout, robust. Corpus bursae somewhat reduced in size.

#### Distribution

**(Fig. 16).** Although we report a few records from central Brazil (Distrito Federal), most records of this species are restricted to the Brazilian Atlantic Forest in the states of Bahia, Espírito Santo, Rio de Janeiro, São Paulo, Paraná, and Santa Catarina. See remarks for potential issues pertaining to the Cerrado records.

#### Remarks.

As mentioned in the remarks of *Tarema rivara*, *Tarema fuscosa* shows a trend in distribution where it is more commonly encountered in humid Atlantic Forest than elsewhere in Brazil. Although we do have some records of *Tarema fuscosa* from Distrito Federal, there is a possibility that they were mislabeled. Out of over 100 examined specimens of *Tarema fuscosa*, the only Cerrado material was from the same collector, who also collected in regions where *Tarema fuscosa* would be more expected, such as the Brazilian states of Paraná and São Paulo. We could not locate any *Tarema fuscosa* specimens from the Cerrado among the Mimallonidae specimens collected there in the USNM, NHMUK, or CPAC.

Prior to this work, the female of *Tarema fuscosa* was not reported in the literature; therefore we describe and figure it here for the first time.

### Tarema bruna sp. n.

Taxon classificationAnimaliaLepidopteraMimallonidae

http://zoobank.org/F649B060-A03F-4CD2-BF63-F35071A6B9A1

Figs 7 , 13 , 16

#### Type material.

**Holotype**, ♂. **BRAZIL: São Paulo**: Alto de [*recte* da] Serra, [Paranapiacaba, Santo André], São Paulo, November, 1922. (R. Spitz [leg.])./ Rothschild Bequest BM 1939-1/ [genitalia] VIAL NHMUK010402168 / NHMUK010318286/ HOLOTYPE male *Tarema bruna* St Laurent, Herbin, & C. Mielke, 2017 [handwritten red label]/ (NHMUK). Type locality: Brazil: São Paulo: Paranapiacaba.

#### Diagnosis.

Externally this species is most similar to *Tarema rivara*, but can be easily distinguished by the earthen brown and clay brown coloration rather than orange or red-orange in *Tarema rivara*. Additionally, in *Tarema bruna* sp. n. the postmedial lunule reaches the wing margin without becoming highly diffuse, and is parallel to the wing margin for its entire length until bending outward. The male genitalia is unique in the extreme reduction of the heavily sclerotized vincular/valve arms, present as a small extension at the base of the valves. The phallus is also unique in the thinness of the dorsal projection, which is smooth as in *Tarema rivara*, not spined as in *Tarema fuscosa*.

#### Description.

**Male.** *Head*: As for genus, coloration earthen brown. *Thorax*: Coloration similar to that of head, appearing hoary due to banded gray and cream colored scales interspersed amongst brown ones, prothorax covered almost entirely in these lighter scales. *Legs*: As for genus. *Forewing dorsum*: Forewing length: 13 mm, wingspan: 26 mm, n=1. Ground color a mixture of earthen brown tones and clay-brown, overall generously shaded by cream colored scales giving the wing a hoary, layered appearance, especially medially. Antemedial line faint, brown, wavy. Postmedial line as for genus but wavier, coloration light cream, not bordered by darker scales except for a small external portion above the tornus. Antemedial area with salmon orange hue, medial area lighter brown compared to darker submarginal area. Apical half of submarginal area with postmedial lunule, the latter parallel with margin, then smoothly curved toward margin reaching wing margin without becoming diffuse, basal half of submarginal area darkest brown, apical portion external to lunule lighter brown. Discal spot as for genus. Fringe light cream with lighter and darker patches. *Forewing ventrum*: Similar to dorsum but lighter due to more extensive covering of gray and cream colored scales; antemedial line absent, postmedial line faint, bulging outward toward wing margin mesally. Postmedial lunule present as on dorsum, more distinct than postmedial line. *Hindwing dorsum*: Coloration as for forewing dorsum, following similar patterning but antemedial line absent, postmedial line straight and faintly outlined by black scales, postmedial lunule very faint. *Hindwing ventrum*: Following same pattern as forewing ventrum, though discal mark very dark, well defined as black oval. *Abdomen*: As for genus, concolorous with thorax. *Genitalia*: (Fig. 13) n=1. Vinculum somewhat ovoid, ventrally with reduced saccus. Uncus robust but reduced to slightly triangular stump. Gnathos a tapered, elongated, hexagonal plate. Valves short, rounded. Base of valves with pair of long, fingerlike projections. Slightly more strongly sclerotized, small projections emanate from base of valves. Diaphragm with pair of horsetail-like setal patches consisting of very long setae that extend outward over phallus below gnathos plate, setae curled backward at end. Phallus broad, large, widened distally, with two elongated accessory projections, one projection superior to phallus, smooth, narrow, pointed, other projection longer, narrower, running laterally along phallus originating from within phallus, tip of second projection sharp, angled forward. Vesica balloon-like. **Female.** Unknown.

#### Distribution

**(Fig. 16).** This new species is so far known only from the type locality at Paranapiacaba (previously known as Alto da Serra, a train station), São Paulo, Brazil. According to GoogleEarth, the elevation at this locality is approximately 700 m.

#### Etymology.

This species is named for its brown (*bruna* Latin) coloration, which largely distinguishes it from the red or orange *Tarema rivara* and the black, gray, and cream-colored *Tarema fuscosa*.

#### Remarks.

The discovery of a unique new species of *Tarema* from eastern São Paulo is surprising because this is a relatively well-surveyed region of Brazil (R. A. St. Laurent pers. obs.). Both *Tarema rivara* and *Tarema fuscosa* have been collected from the type locality of *Tarema bruna* (NHMUK), though at different times of the year. As previously mentioned in the remarks of *Tarema rivara*, that species is primarily a summer species, with records from Paranapiacaba in January, while *Tarema fuscosa* has only been collected there in the winter (June and July). More material of *Tarema bruna* will be needed to verify its voltinism.

An issue is presented by the fact that the type localities of *Tarema rivara*, *Tarema macarina*, and *Tarema bruna* are all from São Paulo, Brazil with specific type locality information from within the state only available for *Tarema bruna*. Therefore, the possibility arose that the name *Tarema macarina* could be wrongfully synonymized with *Tarema rivara* if indeed it is conspecific with the new species described herein. However, we consider the apparent rarity of *Tarema bruna* combined with the genitalia similarities between the lectotype of *Tarema macarina* and other *Tarema rivara* females dissected from São Paulo and elsewhere, including Cerrado regions, more suggestive that the name *Tarema macarina* does in fact represent the female of *Tarema rivara*, a much more commonly collected and widespread species. If future evidence were found to contradict our hypothesis, *Tarema bruna* would then be a junior and subjective synonym of *Tarema macarina*. It is therefore necessary that more material of *Tarema bruna* be found or collected, particularly in aim to locate the female of this species.

## Supplementary Material

XML Treatment for Tarema

XML Treatment for Tarema rivara

XML Treatment for Tarema fuscosa

XML Treatment for Tarema bruna
